# Supplementary material for: Evaluation of a novel quantitative multiparametric MR sequence for radiation therapy treatment response assessment
Source: J Appl Clin Med Phys. 2025 Oct 7;26(10):e70274. doi: 10.1002/acm2.70274 (PMC12504055; doi:10.1002/acm2.70274)
Supplement: Supplementary file 1 — Supporting Information [file ACM2-26-e70274-s001.docx]

To determine the impact of the different HN coils on the quantitative DL-MUPA derived T1 and T2 results, a coil comparison was performed using the same phantom and 1.5T GE MR scanner as described in the Methods. The NIST-ISMRM phantom was scanned with DL-MUPA using the two HN coils (GE GEM RT Open suite and GE AIR Open RT suite). DL-MUPA acquisition was repeated five times consecutively for each coil and the phantom temperature was monitored throughout the data acquisition. T1 and T2 quantifications were analyzed following the same methodology described in the Method section, under Phantom Benchmarking, Image Processing and Quantitative Analysis.

Due to a recent scanner platform upgrade to Version MR30.1 R04, a constancy check was performed by scanning the phantom with the brain coil following the above methodology and data were compared to previous phantom benchmarking. Minimal temperature variations were found within 0.6 °C thus no temperature calibration was deemed necessary. Within physiological ranges (reference T1 [246-1741 ms]), minimal differences were found in T1 quantification (<1.3%) using the brain coil as compared to the prior longitudinal phantom benchmarking. However, for reference T2 values in physiological ranges of 42-493 ms, slightly larger variations in T2 values (up to 7.4%) were observed from the prior measurements and similar magnitude to the intersession CV of up to 6.6% for T2.

Comparing phantom measurements of the two HN coils (e.g., GE GEM RT Open suite and GE AIR Open RT suite) within the same physiological range as depicted in Figure S1, close agreement (<5% difference) and strong linear association (R^2^>0.99) were observed for both T1 and T2. However, larger discrepancies were observed for the T2 vials of ~350 ms and ~30 ms where differences were on the order of ~11%. For a general comparison, our prior phantom benchmarking also demonstrated higher variations for similar T2 values (CV=4.8%-6.6%) as compared with the other T2 vials that exhibited a CV<2.4%. Within the phantom QMRI values similar to that expected for the masseter examined in the HN cohort (T1~900-1100 ms, T2~25 ms), the HN coil comparison showed excellent T1 agreement (<1%) with higher T2 differences (~11%).

While the phantom experiment suggested consistency in T1 values between the two HN coils, further in vivo investigation is warranted. Other studies in the literature have reported mixed results of in vivo QMRI agreement using different coils.^1,2^ Other confounding factors such as correcting for B1 inhomogeneities can be explored in future work to further reduce uncertainties of daily variations in QMRI.^3^

Figure S1. Comparison of DL-MUPA (A) T1 and (B) T2 quantifications using the GE GEM RT Open suite coil (x-axis) and the GE AIR Open RT suite coil (y-axis). The center and width of each cross represent mean and intra-session standard deviation of quantification of each vial, respectively. (C) Different coil configurations used in the phantom study.

**References**

1. Krauss W, Gunnarsson M, Andersson T, Thunberg P. Accuracy and reproducibility of a quantitative magnetic resonance imaging method for concurrent measurements of tissue relaxation times and proton density. *Magn Reson Imaging*. 2015;33(5):584-591. doi:10.1016/j.mri.2015.02.013

2. Panman JL, To YY, Van Der Ende EL, et al. Bias introduced by multiple head coils in mri research: An 8 channel and 32 channel coil comparison. *Front Neurosci*. 2019;13(JUL):460195. doi:10.3389/FNINS.2019.00729

3. Cho J, Gagoski B, Kim TH, et al. Time-efficient, high-resolution 3T whole-brain relaxometry using 3D-QALAS with wave-CAIPI readouts. *Magn Reson Med*. 2024;91(2):630-639. doi:10.1002/MRM.29865
